# Within‐Ecosystem Comparison of Bigmouth Buffalo *Ictiobus cyprinellus* and Common Carp *Cyprinus carpio* Reveals Diverging Population Trajectories, Declining Recruitment, and a Lifespan of 148 Years

**DOI:** 10.1002/ece3.72483

**Published:** 2025-11-16

**Authors:** Alec R. Lackmann, Jeff Sereda, James Villeneuve, Michelle Foley, Mike Pollock, Reid Bryshun, Katlin McCallum, Ethan Englot, Megan Zak, Cole Rehbein, Ewelina S. Bielak‐Lackmann, Mark E. Clark

**Affiliations:** ^1^ University of Minnesota Duluth Department of Biology Duluth Minnesota USA; ^2^ University of Minnesota Duluth, Department of Mathematics and Statistics Duluth Minnesota USA; ^3^ Water Security Agency Moose Jaw Saskatchewan Canada; ^4^ University of Saskatchewan Department of Biology Canada

**Keywords:** buffalofish, Catostomidae, otoliths, periodic strategist, population‐level inference

## Abstract

The bigmouth buffalo *Ictiobus cyprinellus* is a long‐lived, migratory freshwater fish native to North America whose numbers are declining amidst increasing conservation concerns. Recent studies have uncovered long lifespans, delayed maturation, and episodic recruitment of bigmouth buffalo. Building from previous work in the Qu'Appelle watershed of Saskatchewan, here we quantify otolith‐derived population demographics of bigmouth buffalo and invasive common carp *Cyprinus carpio* across multiple sites in the drainage. The common carp (*n* = 125) and bigmouth buffalo (*n* = 173) collected from 2018 to 2024 reveal that common carp reach asymptotic size two times faster, live three times shorter lives, and invest significantly more into reproduction while also exhibiting recruitment stability during the water control era (post‐1958). Indeed, invasive common carp now outnumber native bigmouth buffalo in this watershed by at least an order of magnitude. In contrast, only a single year class (1997) was evident for bigmouth buffalo after 1949. Therefore, only one recruitment year was evident for this species since common carp were first detected in the system in 1955. Remarkably, we find that as of 2024 more than 90% of bigmouth buffalo in this system are greater than 75 years old with a known maximum age of 148 years. We now know that the bigmouth buffalo is the 11th longest‐lived vertebrate out of more than 66,000 species, and across diverse systems can have recruitment gaps longer than any other animal. Bigmouth buffalo require immediate conservation reassessment amidst ongoing population declines.

## Graphical abstract

Within‐ecosystem comparison of invasive common carp and native bigmouth buffalo reveals recruitment gaps for bigmouth buffalo longer than any other animal and lifespans approaching 150 years. In the post‐dam era, invasive common carp are increasing in number while native bigmouth buffalo are declining.

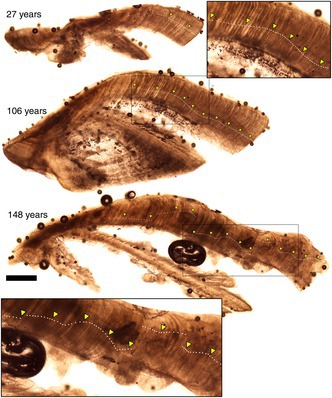

## 1 Introduction

The bigmouth buffalo *Ictiobus cyprinellus* is a long‐lived, migratory freshwater fish native to the Mississippi and Hudson Bay drainages for which some populations are declining in Canada, North Dakota, and Minnesota due to multiple factors including range contraction, habitat fragmentation, water control, invasive species, and unregulated exploitation (Eddy and Underhill 1974; Lackmann et al. 2019, Lackmann et al. 2021, Lackmann, Sereda, et al. 2023, Lackmann, Seybold, et al. 2024, 2025; Gutowsky et al. 2024; Bryshun et al. 2025). It is the largest member of Catostomidae (Eddy and Underhill 1974), a family known for its freshwater diversity and overall imperiled status in North America (Harris et al. 2014). As the only member of Catostomidae that filter feeds on plankton (Johnson 1963), it is uniquely adapted to freshwater ecosystems with some parallels to baleen whales of oceans including filter‐feeding, a relatively large body size within their respective ecosystem, and long lifespan (Mysticeti; Breed et al. 2024). Canada federally listed bigmouth buffalo as a species of special concern under the Species at Risk Act in 2011 (Bigmouth Buffalo Management Plan 2021), a status which it currently maintains. On the other hand, common carp *Cyprinus carpio* (Cyprinidae) is a widespread invasive freshwater fish introduced to North America (Vilizzi 2018) from Eurasia by various fish commissions and private individuals in the 1800s (Hoffbeck 2001). Common carp were first detected in the Qu'Appelle watershed of Saskatchewan (setting of this study) around the year 1955 (Atton 1959).

Despite the federally listed status of bigmouth buffalo in Canada, in the United States bigmouth buffalo harvest is usually regulated collectively with common carp. For example, current fishing regulations in at least 19 of the 22 states in the United States where bigmouth buffalo are endemic, categorize bigmouth buffalo, and common carp in the derogatory “rough fish” group by having no limits on their harvest (Lackmann et al. 2019; Rypel et al. 2021). The “rough fish” label is problematic in a variety of ways (Rypel et al. 2021), but establishing generous harvest regulations (e.g., no limits or seasons in contiguous waters of Minnesota and North Dakota; MNDNR 2025; NDGF 2025) for the category assumes all species and populations in the group can be considered a single stock, that is, with identical population dynamics (Lackmann et al. 2019, 2021; Lackmann, Seybold, et al. 2024; Rypel et al. 2021). This is problematic because several bigmouth buffalo populations are also declining in these areas (Eddy and Underhill 1974; Lackmann et al. 2019, 2021; Lackmann, Seybold, et al. 2024), even as growing, unregulated, and lethal sport fisheries have emerged (Scarnecchia and Schooley 2020; Scarnecchia et al. 2021; Rypel et al. 2021; Lackmann, Watkinson, et al. 2024). Basic limiting of this unprecedented waste (Lackmann, Bielak‐Lackmann, et al. 2024) has been proposed in some states (Montague et al. 2023; MTFWP 2025) including Minnesota (Winter 2024), but implementation remains overdue, including for bigmouth buffalo.

Bigmouth buffalo are long‐lived periodic strategists that can experience episodic recruitment across multidecadal scales (Lackmann et al. 2019, 2021; Lackmann, Sereda, et al. 2023; Lackmann, Seybold, et al. 2024; Kopf et al. 2025). Bigmouth buffalo, like many other long‐lived fishes, mature late (Johnson and Kochinsky 1961; Lackmann et al. 2019, 2021), broadcast spawn, have high fecundity, provide no parental care, and invest little per offspring (Lackmann, Sereda, et al. 2023; Lackmann, Seybold, et al. 2024). These naturally selected traits reflect the many survival challenges of diverse freshwater ecosystems (Winemiller 1992; Winemiller and Rose 1992). In addition, bigmouth buffalo continue to improve physiologically as they approach 100 years of age (Sauer et al. 2021), which suggests they may have a natural lifespan greater than 127 years (Lackmann, Sereda, et al. 2023). Bigmouth buffalo also have been found to accrue black and orange pigmentation markings with age in ecosystems from Minnesota and Arizona, markings which allow for the identification of individuals across time (Lackmann et al. 2019; Lackmann, Black, et al. 2023; Lackmann, Seybold, et al. 2024), much like whales (Breed et al. 2024). Furthermore, habitat fragmentation and water control measures due to dams have likely exacerbated recruitment failure for the species in some ecosystems. For example, increased water level in spring was positively correlated with recruitment success of bigmouth buffalo at Jamestown Reservoir, North Dakota (Lackmann et al. 2021), and evidence of skip‐spawning in low water years has been noted in Canada, as well as failed recruitment if the water level–recession rate post‐spawn is too rapid (Lackmann, Sereda, et al. 2023). Furthermore, habitat fragmentation due to dams in the 1930s and 1950s is coincident with the start of multidecadal gaps in recruitment in northwestern Minnesota (Lackmann et al. 2019) and east central Minnesota (Lackmann, Seybold, et al. 2024), respectively. Despite these gains in knowledge and growing conservation concern, otolith‐derived information on comparative life history metrics of bigmouth buffalo and common carp, one of the most notorious invasive species in North America (Vilizzi 2018), is lacking. Such comparative information is necessary to develop and revise sustainable management plans for bigmouth buffalo because bigmouth buffalo co‐occur with common carp across most of their range (Vilizzi 2018). Furthermore, one hypothesis for the observed decline of bigmouth buffalo in the Qu'Appelle watershed is that the increased abundance of common carp (Goodchild 1989; Bigmouth Buffalo Management Plan 2021) has increased competition for spawning, nursery, and other habitat usage in these shallow lakes (Lackmann et al. 2025).

In this study we assess population characteristics from age estimates derived from otoliths of bigmouth buffalo and common carp from the Qu'Appelle watershed of Saskatchewan. Using age estimates, we model body growth and recruitment patterns of the populations, and make direct comparisons between the two species. We also analyze spot‐pigmentation accrual of bigmouth buffalo from Saskatchewan and compare it to other systems. We place these findings within the larger literature on bigmouth buffalo and their growing need for conservation, as well as the longest‐lived vertebrates.

## 2 Methods

### 2.1 Study Sites

**FIGURE 1:**
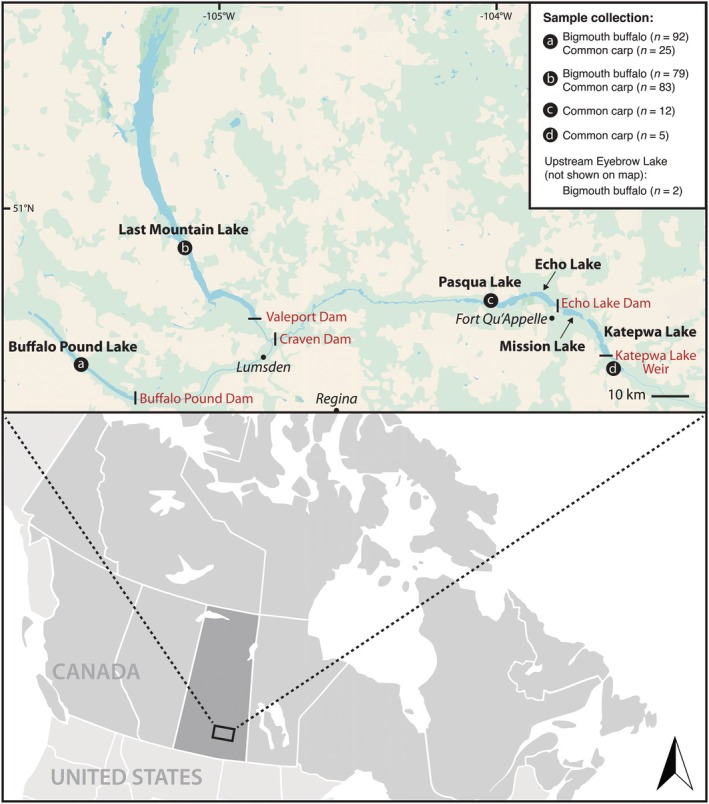
Map of the Qu'Appelle watershed showing sample collections of recruited bigmouth buffalo *Ictiobus cyprinellus* and common carp *Cyprinus carpio* from Saskatchewan during 2018–2024. The major dams or weirs along this reach of the watershed are also noted, and major towns are italicized. Sample size for each site and species is provided in the legend. Note that the Upstream Eyebrow Lake site, which is northwest of Buffalo Pound Lake by about 80 km, is outside the scope of this map but is shown in Lackmann, Sereda, et al. (2023).

Sampling took place within the Qu'Appelle River watershed in Saskatchewan, Canada. The main study lakes included the upper Qu'Appelle lakes of Buffalo Pound Lake (50°39′ N 105°30′ W) and Last Mountain Lake (51°10′ N 105°15′ W) (Figure 1). A small sampling site was also located upstream of Eyebrow Lake near Lake Diefenbaker (Lackmann, Sereda, et al. 2023). Lower Qu'Appelle (downstream) sites included Pasqua Lake (50°46′ N 103°59′ W) and Katepwa Lake Weir (50°41′ N 103°37′ W) (Figure 1). Valeport Dam, a dam that was initially constructed on Last Mountain Lake during 1939–1944, but removed in 1947, and then reconstructed in 1958 (Pasqua First Nation 2012) until its permanent removal in 2022 (J. Sereda personal observations), was built to regulate water levels. Post‐2022, water levels in Last Mountain Lake continue to be regulated via Craven Dam (reconstructed in 2003 (Pasqua First Nation 2012)) (Figure 1).

### 2.2 Sample Collection

Fish were collected from 2018 to 2024 across the sampling sites. For sample collection information from 2018 to 2021 for bigmouth buffalo (*n* = 52) and common carp (*n* = 17) see Lackmann, Sereda, et al. (2023). From 2022 to 2024, more bigmouth buffalo (*n* = 121) and common carp (*n* = 108) were collected. Specifically, these additional 2022–2024 common carp were collected from Buffalo Pound Lake (*n* = 5), Katepwa Lake Weir (*n* = 5), Last Mountain Lake (*n* = 78), and Pasqua Lake (*n* = 12) in 2022; from Buffalo Pound Lake (*n* = 3) in 2023; and from Last Mountain Lake in 2024 (*n* = 5). The additional 2022–2024 bigmouth buffalo were collected from Buffalo Pound Lake in 2022 and 2023 (*n* = 6; *n* = 36, respectively), and Last Mountain Lake in 2022, 2023, and 2024 (*n* = 37; *n* = 2; *n* = 40, respectively). Overall, fish were collected using a variety of nets and gears throughout the entire growing season, designed to catch the entire range of fish sizes. Namely, we collected bigmouth buffalo and common carp using a variety of gill nets including (60 m long × 1.8 m deep, and consisting of 10 m panels each of 3.8, 5.1, 7.6, 10.2, 12.7 and 14.0 cm stretched multifilament mesh; 60 m long and 22 cm mesh across; and 100 m long 22 cm mesh across), seines (30 m long × 1 cm mesh), beamish traps (30 m lead, 1 m deep, 0.5 cm mesh), and downhaul traps (50 m lead, 2 m deep, 3 cm mesh). All captured bigmouth buffalo were retained for analysis, except after 2023 in Buffalo Pound Lake, where collection of bigmouth buffalo was discontinued because the species is federally listed as a species of special concern under the Species at Risk Act, which restricts collection (Lackmann, Sereda, et al. 2023; J. Sereda and Saskatchewan Ministry of Environment, personal communication 2024). Common carp were selectively retained to capture a representative sample as they are abundant across all sampling sites and outnumbered captures of bigmouth buffalo by at least an order of magnitude.

In addition, we collected young‐of‐the‐year common carp and bigmouth buffalo and measured them for total length (±1 mm). A subsample of young‐of‐the‐year common carp, which outnumber captures of bigmouth buffalo young‐of‐the‐year by more than an order of magnitude, was collected (*n* = 31) on 28 July 2022 (~2‐month‐old) from the Katepwa Lake Weir. We also systematically sampled from 1 to 10 August 2023 for young‐of‐the‐year bigmouth buffalo (~3‐month‐old) when 27 individuals were collected and measured. Specifically, we collected young‐of‐the‐year bigmouth buffalo from Katepwa Lake (*n* = 4), Buffalo Pound Lake (*n* = 2), Mission Lake (*n* = 5), Crooked Lake (*n* = 1), and Pasqua Lake (*n* = 15).

### 2.3 Body Dissections

For the non‐young‐of‐the‐year fish, we measured the size of individuals and extracted otoliths for later use in estimating age. We quantified size by wet mass (±0.01 kg) and total length (±1 mm) immediately after the fish was collected and then dissected gonadal tissue to determine sex. We also began measuring gonad mass (±0.01 g) in 2021, and we were able to do so for 113 of 114 common carp and 122 of 128 bigmouth buffalo collected post‐2020. After dissection, we removed the head of the fish for storage for otolith extraction later in the lab. We extracted otoliths following Lackmann et al. (2019), (Lackmann et al. 2021). We also assessed all bigmouth buffalo collected for the presence or absence of black or orange pigmentation spots following the protocol of Lackmann et al. (2019), (Lackmann, Black, et al. 2023, Lackmann, Seybold, et al. 2024), except for aged individuals collected in 2018 (*n* = 11), nonphotographed individuals in 2020 (*n* = 19 of 34), and 1 individual from 2024 that was excessively battered from collection gear. All adult bigmouth buffalo were assessed for white‐edged fins (Lackmann, Seybold, et al. 2024).

### 2.4 Otolith Analysis

In the lab we processed extracted otoliths to obtain photographs of their whole structure and thin‐sectioned them to determine age. For these detailed Methods see Lackmann et al. (2019) and (Lackmann et al. 2021, Lackmann, Sereda, et al. 2023). In summary, asteriscus or lapillus otoliths (these are the largest otoliths in cypriniform fishes) were embedded in epoxy and thin‐sectioned along the primary growth axis using a low‐speed saw equipped with twin diamond‐embedded blades separated by a spacer (Lackmann et al. 2019). Resulting otolith thin sections were then immersed in mineral oil and photographed using compound microscopy and transmitted light, and then digitally assessed and age‐scored by multiple readers following the age‐validated protocol of Lackmann et al. (2019) and (Lackmann et al. 2021). The otoliths of bigmouth buffalo and other buffalofish species have been age‐validated several times (Lackmann et al. 2019, 2021; Long et al. 2023). For bigmouth buffalo, otoliths have been age‐validated latitudinally across individuals (Lackmann et al. 2019), longitudinally within old‐age individuals (Lackmann et al. 2019), and longitudinally within young individuals (Lackmann et al. 2021), thereby following methods for comprehensive age validation outlined by Campana (2001). Otoliths have also been age‐validated from common carp (Brown et al. 2004), and longevity up to 64 years from Minnesota has been reported (Dauphinais et al. 2018). We assigned ages and year classes to fish following Lackmann et al. (2019), (Lackmann, Sereda, et al. 2023).

### 2.5 Statistical Analysis

We quantified age score precision via the coefficient of variation (CV) (Campana et al. 1995), and quantified episodicity of recruitment for bigmouth buffalo and common carp using a likelihood ratio test. Using sample data and following Lackmann et al. (2021) and (Lackmann, Sereda, et al. 2023, Lackmann, Seybold, et al. 2024), we defined “evidence of recruitment” categorically (Yes/No) for each year 1876–2023, based on whether a 2018–2024 collected bigmouth buffalo was from that respective year class. We used 1876 as the starting point because that is the earliest year class represented in the sample. We followed the same method for common carp but used 1967 as the starting point because that is the earliest year class in that sample. We used contingency analysis to test whether evidence of recruitment was distributed randomly or episodically (i.e., less likely to be observed if recruitment was not observed in the previous year). We ran this analysis for each species pooling sites, and again for each species in each of the two main study lakes (Buffalo Pound Lake, Last Mountain Lake). We used a chi‐square test for a single variable to test whether the two most distinct modal year classes (1948 and 1997) were significantly different in their relative proportion evident in each of the two main study lakes. We also used contingency analysis to test if bigmouth buffalo recruitment was significantly different in the pre‐common carp era (prior to 1955) compared to the post‐common carp era in Last Mountain Lake, as well as in the pre‐Valeport‐dam‐reconstruction era (prior to 1958) compared to the post‐Valeport‐dam‐reconstruction era, and comparing the pre‐carp/pre‐Valeport‐dam‐reconstruction era versus the post‐carp/pre‐Valeport‐dam‐reconstruction period of 1955–1958.

Catch‐curve analysis (Maceina 1997) was performed on common carp collected from Last Mountain Lake and used to estimate the annual mortality rate *A* and subsequently analyze recruitment patterns in correlation to spring water levels. The ascending limb of the catch curve was excluded in the calculation of *A*, as is routine (Smith et al. 2012). Catch‐curve analysis (and hydrological analysis, see later in paragraph) was not performed for common carp collected from Buffalo Pound Lake due to insufficient sample size, and was not completed for bigmouth buffalo because only 1 year class is evident (1997) since the late 1940s in this system (consistent hydrometric data collection began in the 1950s). We then used the estimate of *A* for common carp in Last Mountain Lake, to standardize year class count via the formula: $\text{Aycc}=\mathrm{ycc}{\left(\frac{1}{1-A}\right)}^{\mathrm{Age}}$ where Aycc is the *A*‐standardized year class count, ycc is a given year class count observed in the sample, *A* is the annual mortality rate, and Age is the age of the cohort as of 2024. We used linear regression analysis to assess whether the standardized year class count for common carp was correlated with spring peak water level in Last Mountain Lake. Monthly or semi‐monthly lake elevation data were available for Last Mountain Lake from 1944 to 1952, and approximately daily from 1953 to 2023 and were used to quantify spring water levels. The potential relationship between hydrology and bigmouth buffalo recruitment in Last Mountain Lake could not be analyzed because of the relative lack of water‐level data prior to the Valeport dam reconstruction in 1958, and due to the paucity of bigmouth buffalo recruitment since 1949. For analysis of spawning and recruitment of bigmouth buffalo from Buffalo Pound Lake in the water‐level‐data era (starting in 1955), see Lackmann, Sereda, et al. (2023).

We used the von Bertalanffy growth function (von Bertalanffy 1938) to model size at age for each species such that $\mathrm{TL}=L_{\infty }\cdot \left(1-e^{-k\cdot \left(\mathrm{age}-t_{0}\right)}\right)$, where TL is total length (cm), age is in years, *L*_*∞*_ is asymptotic total length (cm), parameter *k* is the instantaneous rate of increase (cm/cm/d) (Schnute and Fournier 1980), and parameter *t*_0_ is age (years) at length 0. We constrained the von Bertalanffy models for bigmouth buffalo such that TL was 1.9 cm (males and females) at age 1/12 year (based on the observed size of month‐old fry—see Lackmann, Sereda, et al. 2023) through the *t*_*0*_ parameter with $t_{0}=\left(1/k\right)\cdot \left(\ln\left(\left(L_{\infty }-1.9\right)/L_{\infty }\right)\right)-(1/12)$. We excluded from model selection models with a uniform *L*_*∞*_ (not sex‐specific) because bigmouth buffalo are sexually dimorphic at asymptotic size (Lackmann et al. 2019, 2021; Lackmann, Sereda, et al. 2023). We constrained the von Bertalanffy models for common carp such that TL was 4.0 cm (males and females) at age 2/12 year (based on the observed size of ~2‐month‐old young‐of‐the‐year) through the *t*_*0*_ parameter with $t_{0}=\left(1/k\right)\cdot \left(\ln\left(\left(L_{\infty }-4.0\right)/L_{\infty }\right)\right)-(1/6)$. We used information‐theoretic methods (Burnham and Anderson 2002) to determine the highest ranked models for size at age based on the relative Akaike's Information Criterion corrected for small sample sizes (ΔAICc) when comparing multiple models in a suite (Akaike 1973). We ran these analyses for each species pooling across sites, and then again within each of the two primary study lakes. We then compared parameters and their 95% confidence intervals. We could not run this analysis solely for common carp in Buffalo Pound Lake due to insufficient sample size.

We used a two‐sample *t* test to compare pre‐spawn gonadosomatic indices (GSI) of sexually mature female bigmouth buffalo versus sexually mature female common carp. On the basis of previously observed spawning phenologies of both species in this system (Lackmann, Sereda, et al. 2023) and previously observed post‐spawn GSI values (Lackmann, Seybold, et al. 2024), we defined May‐collected bigmouth buffalo with GSI > 0.125 as pre‐spawn, and June‐collected common carp with a GSI > 0.125 as pre‐spawn. For this analysis, all bigmouth buffalo were older than 26 years, longer than 73.5 cm TL, and heavier than 6.49 kg, and all common carp were older than 11 years, longer than 67.9 cm TL, and heavier than 5.01 kg; all individuals were sexually mature. Furthermore, when assessing the distribution of GSI for each species in each respective month, each distribution was bimodal and the break dividing modes occurred at approximately 0.125 GSI, which was consistent with pre‐ versus post‐spawn levels previously documented across the spawning phenology (Lackmann, Seybold, et al. 2024). We did not assess this for males since males invest significantly less in gonads, and the distinction between pre‐ and post‐spawn males (in terms of GSI) is subtle (Lackmann, Seybold, et al. 2024).

We used logistic regression analysis to model the presence (or absence) of black or orange pigmentation spots versus age for bigmouth buffalo. Pigmentation markings have been found to accrue with age in other bigmouth buffalo systems such as in Minnesota (Lackmann et al. 2019; Lackmann, Seybold, et al. 2024) and Arizona (Lackmann, Black, et al. 2023). We used JMP 16 Pro Statistical Discovery for statistical analysis and graphical output.

### 2.6 Ethics Statement

We have treated all animals in accordance with the Water Security Agency and the University of Saskatchewan's Institutional Animal Care and Use Committee (IACUC) guidelines on animal care as specified in each protocol (IACUC protocol 20170034). The collection of specimens was conducted in accordance with all applicable laws, guidelines, and regulations. Specimen collection permits for this study were granted by the Saskatchewan Ministry of Environment (permit numbers: INV05‐SCP2020, INV02A‐SCP2021, INV07A‐SCP2022, INV01‐SCP2023, INV03‐SCP2024).

## 3 Results

### 3.1 Age and Recruitment Analysis

**FIGURE 2:**
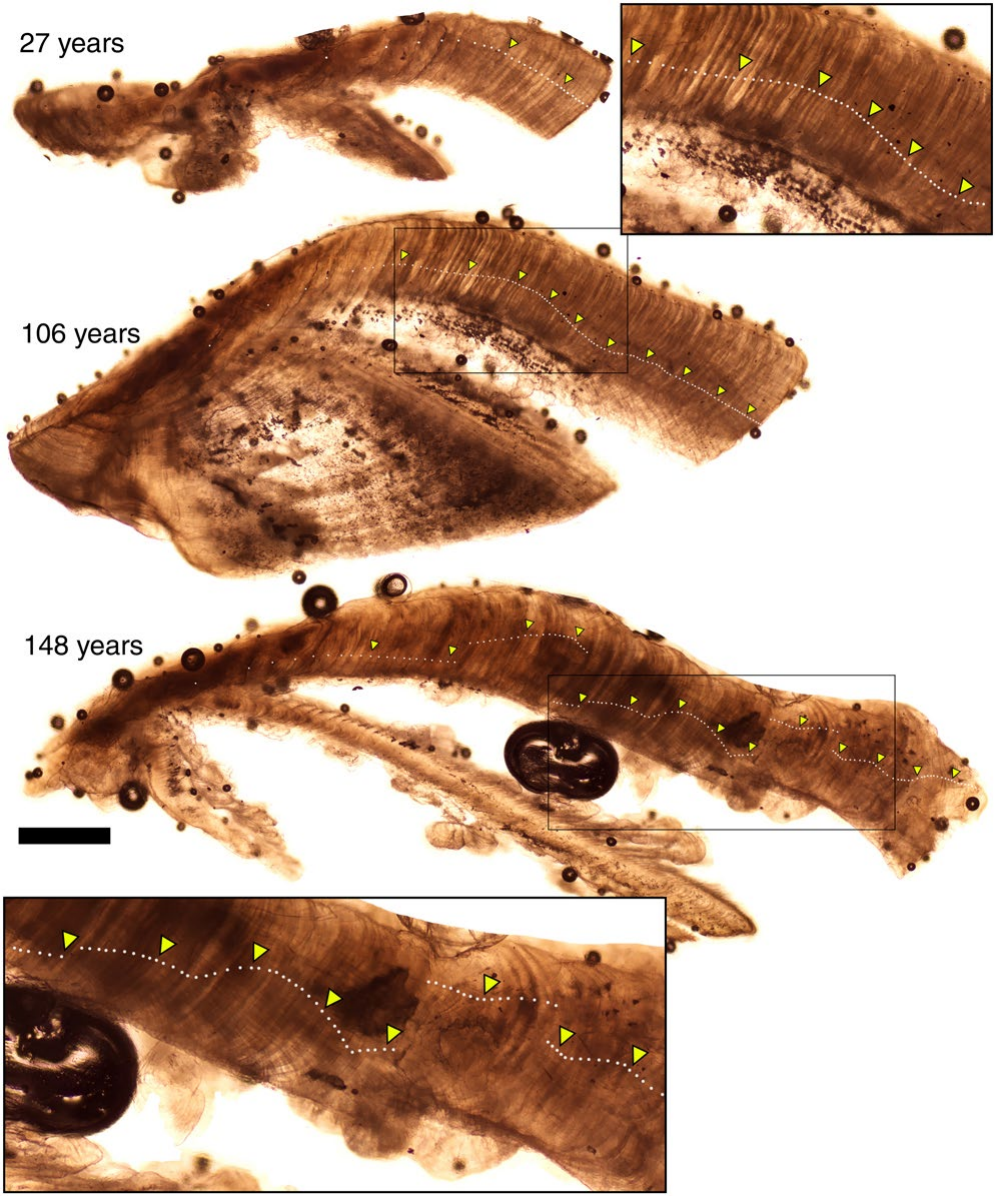
Thin‐sectioned asteriscus otoliths of bigmouth buffalo *Ictiobus cyprinellus* from Last Mountain Lake, Saskatchewan. Examples include a 27‐, 106‐, and 148‐year‐old bigmouth buffalo. Otolith insets show a closeup of approximately 55 years of growth in the 106‐year‐old, and approximately 80 years of growth in the 148‐year‐old. Dots mark each annulus; triangles mark each decade. Scale bar = 600 μm (does not apply to otolith insets).

**FIGURE 3:**
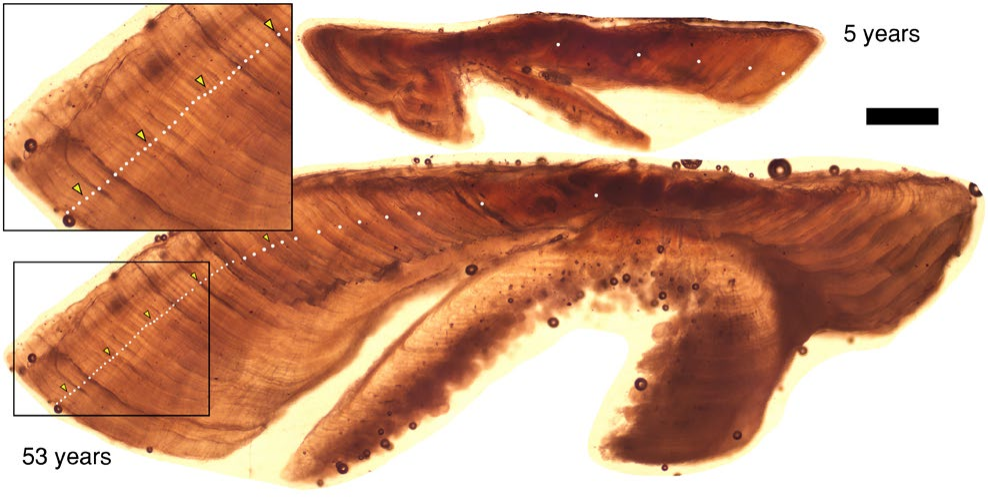
Thin‐sectioned asteriscus otoliths of common carp *Cyprinus carpio* from Buffalo Pound Lake, Saskatchewan. Examples include a 5 and a 53‐year‐old common carp. Otolith inset shows a closeup of approximately 35 years of growth in the 53‐year‐old common carp. Dots mark each annulus; triangles mark each decade. Scale bar = 600 μm (does not apply to otolith inset).

We measured the size and estimated the ages of 173 bigmouth buffalo and 125 common carp in this study. The size of bigmouth buffalo ranged from 62.3 to 101.5 cm TL (interquartile range [IQR] from 73.0 to 84.0 cm, median 77.0 cm) and 4.00 to 17.66 kg (IQR from 6.74 to 10.50 kg, median 7.81 kg), and there were 87 females, 83 males, and 3 unsexed individuals. The size of common carp ranged from 26.5 to 92.0 cm TL (IQR from 64.0 to 75.0 cm, median 69.0 cm) and 0.28 to 16.22 kg (IQR from 3.32 to 7.72 kg, median 4.05 kg), and there were 59 females, 60 males, and 6 individuals for which sex was not determined. In addition, the ~2‐month‐old young‐of‐the‐year common carp (*n* = 31) collected on 28 July 2022 ranged from 3.4 to 4.5 cm TL (IQR from 3.6 to 4.1 cm TL, median 4.0 cm). The ~3‐month‐old young‐of‐the‐year bigmouth buffalo (*n* = 27) collected during 1–10 August 2023 ranged from 5.5 to 10.5 cm TL (IQR from 7.3 to 9.5 cm TL, median 8.2 cm). Excluding the young‐of‐the‐year, which were known‐age, the overall between‐reader aging precision for bigmouth buffalo and common carp had a median coefficient of variation (CV) of 3.2% and 5.9%, respectively (mean of 3.5% and 7.0%, respectively). Overall, across the 7 years of collection (2018–2024), ages ranged from 21 to 148 years old for bigmouth buffalo (e.g., Figure 2), and 3–53 years for common carp (e.g., Figure 3). More than 90% (156 of 173) of bigmouth buffalo were older than 70 years of age, whereas more than 70% (88 of 125) of common carp were younger than 15 years of age.

**FIGURE 4:**
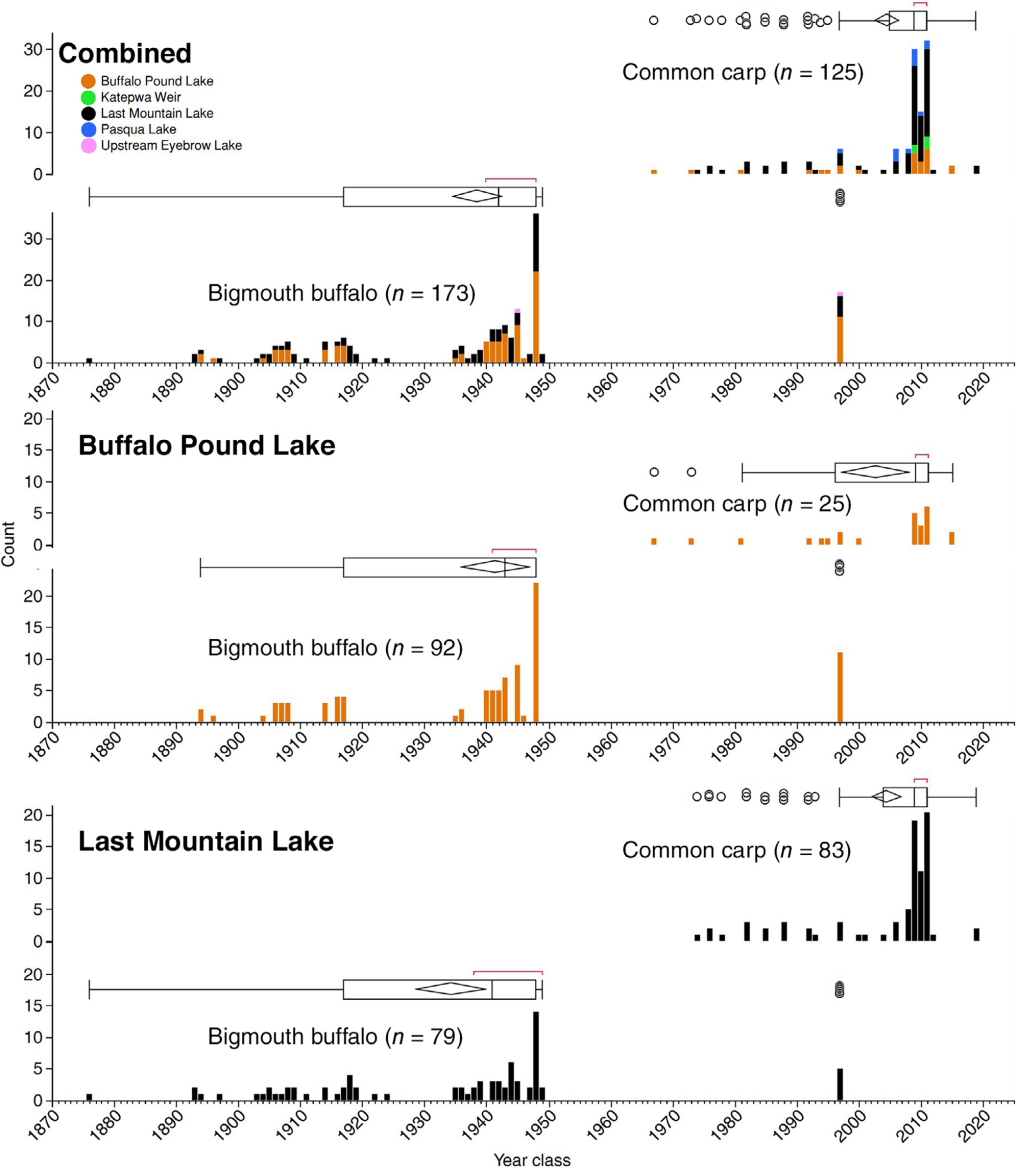
Year class distribution of common carp *Cyprinus carpio* (*n* = 125) versus bigmouth buffalo *Ictiobus cyprinellus* (*n* = 173) collected from the Qu'Appelle watershed, Saskatchewan during 2018–2024, pooling across five (color coded) sites, and in Buffalo Pound and Last Mountain Lake specifically. Year classes ranged from 1967 to 2019 for common carp, and from 1876 to 1997 for bigmouth buffalo. The modal recruited class was in year 2011 for common carp, and year 1948 for bigmouth buffalo. Overall, 90.2% of collected bigmouth buffalo pre‐date the invasion of common carp (~1955) in this system (Atton 1959), and 70.4% of collected common carp were produced after 2007. Box plots are shown above each distribution, with outliers (open black circles) if evident, mean diamonds, and red brackets indicating the shortest half of the data.

We calculated year classes for all fish aged by otolith and found significant differences between the species. With sites pooled, bigmouth buffalo year classes ranged from 1876 to 1997, with 21% of the fish coming from the modal 1948 year class and 90% of individuals from a hatch year before 1949 (Figure 4). On the other hand, common carp year classes ranged from 1967 to 2019, with 62% belonging to the 2009, 2010, or 2011 year classes. While 0% of the bigmouth buffalo were from a year class during the 2000s, more than 78% of the common carp were. Furthermore, evidence of bigmouth buffalo recruitment across years (Yes or No) was episodic, both pooling sites (*χ* ^2^ = 52.1, df = 1, *n* = 147, *p* < 0.0001, *R* ^2^ = 0.29), and within each of the two main study lakes of Buffalo Pound (*χ* ^2^ = 17.3, df = 1, *n* = 129, *p* < 0.0001, *R* ^2^ = 0.13) and Last Mountain (*χ* ^2^ = 35.6, df = 1, *n* = 147, *p* < 0.0001, *R* ^2^ = 0.20). In contrast, a likelihood ratio test indicated that evidence of common carp recruitment was not episodic, both pooling sites (*χ* ^2^ = 0.2, df = 1, *n* = 55, *p* = 0.6196, *R* ^2^ = 0.00), and within each of the two main study lakes of Buffalo Pound (*χ* ^2^ = 0.2, df = 1, *n* = 55, *p* = 0.6243, *R* ^2^ = 0.00) and Last Mountain (*χ* ^2^ = 0.1, df = 1, *n* = 48, *p* = 0.7624, *R* ^2^ = 0.00). Across these sites in the Qu'Appelle watershed, 99.2% (124 of 125) of common carp are from recruited classes in the recent 50‐year period, while only 9.8% of bigmouth buffalo are from a year class in the past 75 years (Figure 4).

Overall, across these sites in the Qu'Appelle watershed, there exist multidecadal periods with no evidence of bigmouth buffalo recruitment. We first assessed the year‐class structure of bigmouth buffalo specifically in each of the two lakes, and the prevalence of both the 1948 and 1997 year classes in Buffalo Pound (23.9%, 12.0%, respectively) was proportionally higher than in Last Mountain (17.7%, 6.3%, respectively). However, these differences were not significant (*p* = 0.1214, *p* = 0.0778, respectively). With a sample size of 173 bigmouth buffalo when pooling these sites, four multidecadal periods exist with no evidence of recruitment: 1877–1892, 1925–1934, 1950–1996, and 1998 to present (Figure 4). While there is only 1 evident recruitment year post‐1949 in the bigmouth buffalo sample (*n* = 173), there are 25 evident recruitment years post‐1967 in the common carp sample (*n* = 125). Indeed, contingency analysis from Last Mountain Lake revealed that bigmouth buffalo recruitment in the pre‐common carp era (prior to 1955) was significantly more likely than in the post‐common carp era (*χ* ^2^ = 32.0, df = 1, *n* = 147, *p* < 0.0001, *R* ^2^ = 0.25). In Last Mountain Lake, contingency analysis also revealed that bigmouth buffalo recruitment in the era before Valeport dam was reconstructed (prior to 1958), was significantly more likely than in the era after Valeport dam was reconstructed (*χ* ^2^ = 29.3, df = 1, *n* = 147, *p* < 0.0001, *R* ^2^ = 0.23). However, bigmouth buffalo recruitment in the pre‐carp/pre‐Valeport‐dam‐reconstruction era was not significantly more likely than in the post‐carp/pre‐Valeport dam era of 1955–1958 (*χ* ^2^ = 2.0, df = 1, *n* = 82, *p* = 0.1580, *R* ^2^ = 0.03).

**FIGURE 5:**
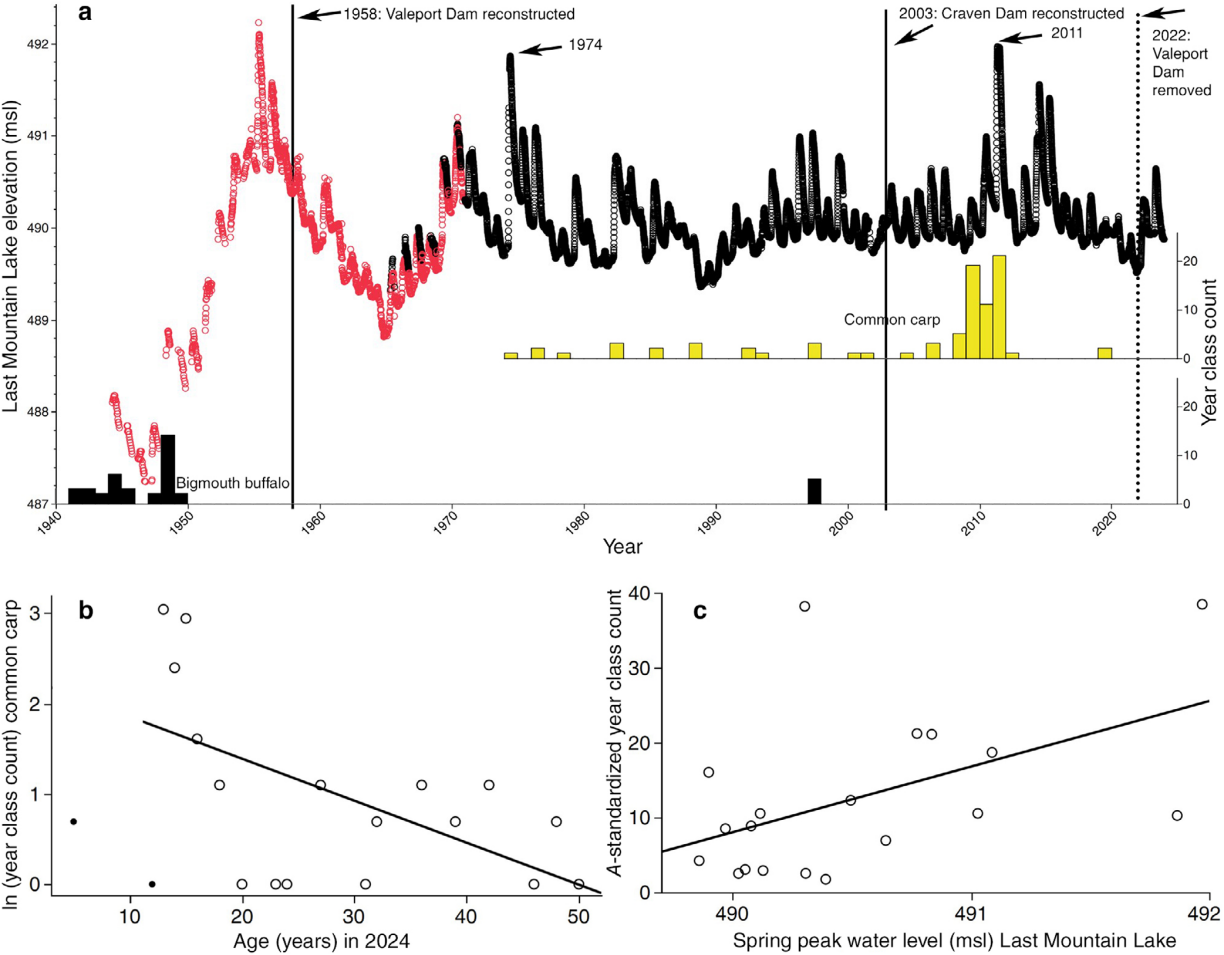
Last Mountain Lake elevation versus recruitment of common carp *Cyprinus carpio* and bigmouth buffalo *Ictiobus cyprinellus* . (a) Red points represent water level data collected from Regina Beach, whereas black points represent water level data collected from Rowan's Ravine, also a site on Last Mountain Lake. Valeport Dam, which controlled water level on Last Mountain Lake, was initially constructed from 1939 to 1944, removed in 1947, and rebuilt in 1958 until its removal in 2022, and since, Craven Dam controls water level on Last Mountain Lake (see Section 2). Water level data was not collected until 1944. Year class distributions across this timeframe are also provided for each species (*y*‐axes on right), but note that 49.4% of bigmouth buffalo in Last Mountain Lake are from year classes that pre‐date 1940 (not shown on this graph). Years post‐dam reconstruction with a spring peak water level > 491.6 m are labeled. (b) Catch‐curve analysis of common carp from Last Mountain Lake (*F*_1,15_ = 7.4, *p* = 0.0157, *R* ^2^ = 0.33) revealed an annual mortality rate *A* = 4.6%. Solid black points (shown for clarity) make up the ascending limb of the catch curve, and were excluded from catch‐curve analysis as is routine (Smith et al. 2012). When the ascending limb is included, catch‐curve analysis of common carp from Last Mountain Lake (*F*_1,17_ = 73.3, *p* = 0.0860, *R* ^2^ = 0.16) revealed an annual mortality rate *A* = 2.9%, but this was not significant. **c** Common carp year class count (standardized by *A*; see Section 2) was positively correlated with spring peak water level in Last Mountain Lake (*F*_1,17_ = 5.5, *p* = 0.0318, *R* ^2^ = 0.24).

The amount of common carp recruitment in Last Mountain Lake is positively correlated with spring water level peak. A total of 83 common carp were collected from Last Mountain Lake, spread across 19 year classes since 1974 (Figure 5a). This is in stark contrast to bigmouth buffalo, for which a total of 79 individuals have been collected for age analysis from this lake, with only 1 year class evident since 1949 (Figure 5a). Catch‐curve analysis of common carp from Last Mountain Lake indicated an annual mortality rate *A* = 4.6% (*F*_1,15_ = 7.4, *p* = 0.0157, *R* ^2^ = 0.33) (Figure 5b), and common carp year class count (standardized by *A*; see Section 2) was positively correlated with spring peak in water level (*F*_1,17_ = 5.5, *p* = 0.0318, *R* ^2^ = 0.24) (Figure 5c). Catch‐curve analysis could not be completed for bigmouth buffalo because only 1 year class is evident in the past 75 years.

### 3.2 Age and Body Growth

**FIGURE 6:**
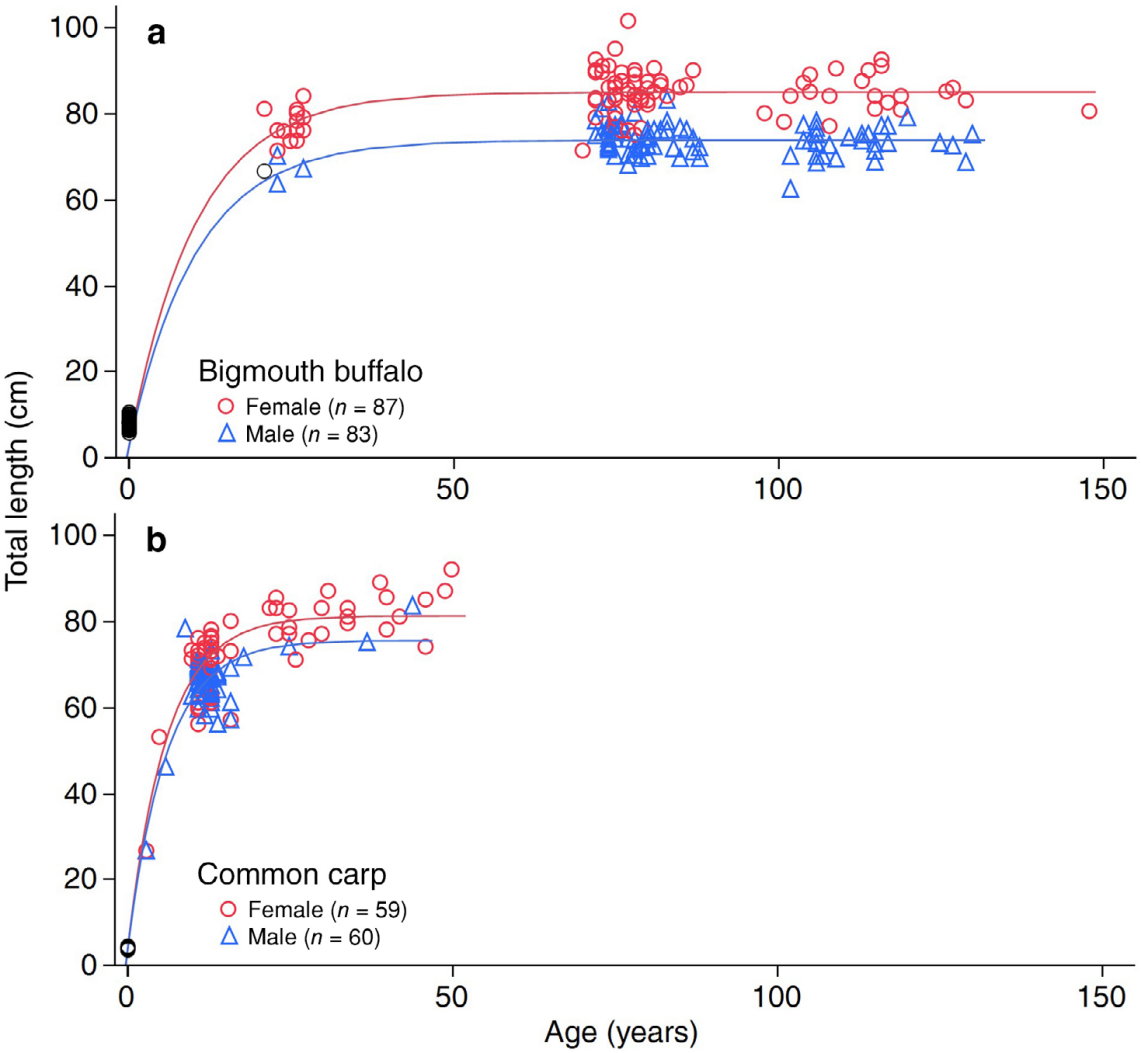
Total length versus age of bigmouth buffalo *Ictiobus cyprinellus* and common carp *Cyprinus carpio* pooled across sites in the Qu'Appelle watershed. (a) Total length versus age of bigmouth buffalo from the Qu'Appelle watershed, Saskatchewan (*F*_3,166_ = 3355.3, df = 3, *p* < 0.0001, *R* ^2^ = 0.98) with asymptotic length (*L*_*∞*_) for females of *L*_*∞*_ = 84.9, 95% CI [83.9, 85.9] and males *L*_*∞*_ = 73.7 [72.4, 75.1], growth rate (*k*) was *k* = 0.097 [0.086, 0.112], and the age at 0 length parameter [*t*_0_] was constrained by 1‐month‐old fry (see Section 2). Unsexed individuals (open black circles; *n* = 28) are plotted for clarity, but did not contribute to the model. (b) Total length versus age of common carp from the Qu'Appelle watershed, Saskatchewan (*F*_3,115_ = 1849.9, df = 3, *p* < 0.0001, *R* ^2^ = 0.97) with asymptotic length (*L*_*∞*_) for females of *L*_*∞*_ = 81.3, 95% CI [78.9, 83.7] and males *L*_*∞*_ = 75.5 [73.1, 78.0], growth rate (*k*) was *k* = 0.167 [0.148, 0.191], and the age at 0 length parameter [*t*_0_] was constrained by 2‐month‐old fry (see Section 2). Unsexed individuals (open black circles; *n* = 31) are plotted for clarity, but did not contribute to the model. See Tables S1 and S2 for model selection statistics for both species, respectively. Points are coded by sex (red line = female model, blue line = male model).

Both bigmouth buffalo and common carp from the Qu'Appelle system are sexually size dimorphic and exhibit pronounced asymptotic growth, but our findings demonstrate that common carp approach asymptotic size approximately two times faster and live significantly shorter lives than bigmouth buffalo. In the highest‐ranked von Bertalanffy growth model for bigmouth buffalo pooling sites (Figure 6, Table S1), the estimated *L*_*∞*_ was 84.9 cm TL for females compared to 73.7 cm for males, and there was no difference in the instantaneous rate of increase (*k* = 0.097) with *t*_*0*_ constrained by month‐old fry (see Section 2). In comparison, common carp exhibited an *L*_*∞*_ of 81.3 cm for females compared to 75.5 cm for males, and there was also no sex‐specific difference in the instantaneous rate of increase (*k* = 0.167) with *t*_*0*_ constrained by 2‐month‐old fry (Figure 6, Table S2; see Section 2). According to these models, bigmouth buffalo females are significantly larger than common carp females, but common carp males overlap in asymptotic length with bigmouth buffalo males. The same patterns are evident (bigmouth buffalo females larger than common carp females, males of both species overlapping in asymptotic size, *k* is approximately two times greater for common carp than it is for bigmouth buffalo) when comparing the two species specifically within Last Mountain Lake (Table 1, Figures S1 and S2; Buffalo Pound Lake did not have sufficient sample size of common carp for modeling length at age). According to these Last Mountain Lake models, common carp reach 95% of asymptotic length by age 17 years, whereas bigmouth buffalo reach the same threshold at approximately 34 years of age (Table 1). Therefore, common carp can live for another 35 years at approximate asymptotic size, whereas bigmouth buffalo can live for more than a century beyond this threshold (Figure 6). When assessing bigmouth buffalo length at age within each of the two study lakes, none of the von Bertalanffy growth model parameters significantly differed (95% CIs overlap for each parameter; Table 1).

**TABLE 1:** Literature review of the highest ranked von Bertalanffy growth function (VBGF) parameter estimates for bigmouth buffalo *Ictiobus cyprinellus* and common carp *Cyprinus carpio* from Saskatchewan, with additional context for each VBGF sample.

Parameter	Lackmann, Sereda, et al. 2023	This study–BPL	This study–LML	This study–LML	This study–Pooled	This study–Pooled
Species	Bigmouth buffalo	Bigmouth buffalo	Bigmouth buffalo	Common carp	Bigmouth buffalo	Common carp
Location	Buffalo Pound Lake	Buffalo Pound Lake	Last Mountain Lake	Last Mountain Lake	BPL, LML, QR	LML, BPL, KW, PL
*N* (♀, ♂)	(32, 17)	(53, 36)	(33, 46)	(35, 48)	(87, 83)	(59, 60)
Age span in years (♀, ♂)	(0–127, 0–114)	(0–127, 0–127)	(0–148, 0–130)	(0–50, 0–44)	(0–148, 0–130)	(0–50, 0–44)
Maximum observed age (years)	127	127	148	50	148	50
Largest YC gap (yrs) within span (♀, ♂)	(49, 49)	(49, 49)	(49, 48)	(8, 12)	(49, 48)	(6, 12)
2nd largest YC gap (yrs) (♀, ♂)	(24, 24)	(27, 27)	(27, 27)	(6, 7)	(27, 27)	(5, 7)
*♀k* (95% CI)	0.107 (0.085, 0.153)	0.103 (0.087, 0.128)	0.089 (0.075, 0.113)	0.170 (0.155, 0.188)	0.097 (0.086, 0.112)	0.167 (0.148, 0.191)
*♂k* (95% CI)	0.107 (0.085, 0.153)	0.103 (0.087, 0.128)	0.089 (0.075, 0.113)	0.170 (0.155, 0.188)	0.097 (0.086, 0.112)	0.167 (0.148, 0.191)
*♀L*_***∞***_ (95% CI)	83.4 (81.5, 85.3)	84.3 (82.9, 85.6)	86.1 (84.6, 87.5)	82.8 (81.1, 84.5)	84.9 (83.9, 85.9)	81.3 (78.9, 83.7)
*♂L*_***∞***_ (95% CI)	73.2 (70.1, 76.3)	73.0 (71.0, 75.0)	74.4 (72.5, 76.2)	76.4 (74.4, 78.4)	73.7 (72.4, 75.1)	75.5 (73.1, 78.0)
*t*_*0*_ (95% CI)	Constrained^a^	Constrained^a^	Constrained^a^	Constrained^a^	Constrained^a^	Constrained^a^
~ Age at estimated 95% of *L*_***∞***_ (♀, ♂)	(28, 28)	(29, 29)	(34, 34)	(17, 17)	(31, 31)	(18, 18)

*Note:* YC, year class; “a” see Section 2.

Abbreviations: BPL, Buffalo Pound Lake; KW, Katepwa Weir; LML, Last Mountain Lake; PL, Pasqua Lake Age 0 individuals are unrecruited 1‐month‐old fry (bigmouth buffalo), or unrecruited 2‐month‐old fry (common carp) used to constrain *t*_*0*_ (*n* values of VBGF do not include young‐of‐the‐year; QR, Upstream of Eyebrow Lake, Qu'Appelle River; see Section 2).

**FIGURE 7:**
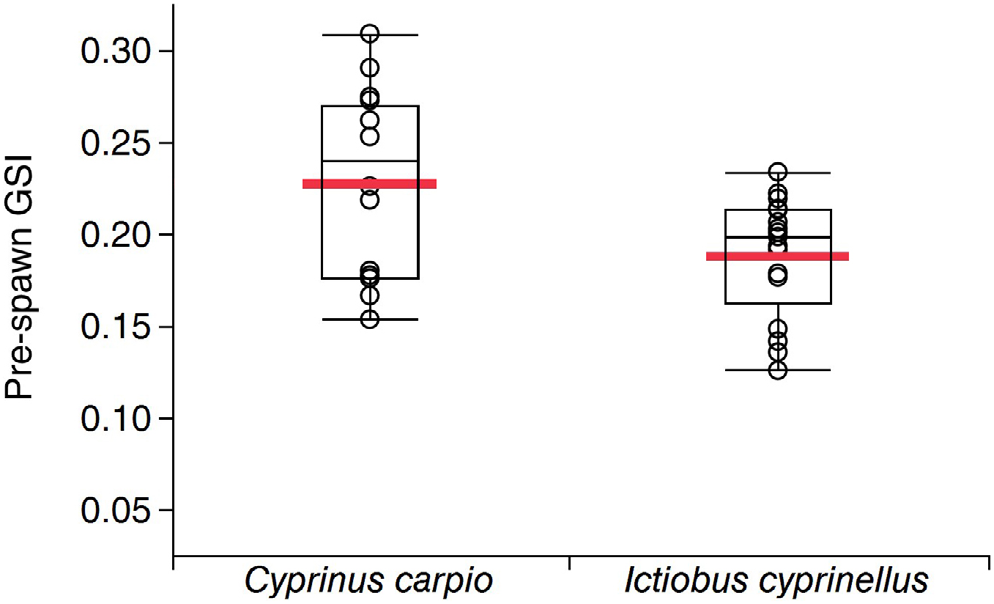
Gonadosomatic index (GSI) of mature pre‐spawn female common carp *Cyrinus carpio* (*n* = 16) versus mature pre‐spawn female bigmouth buffalo *Ictiobus cyprinellus* (*n* = 17) from the Qu'Appelle watershed, Saskatchewan. On average, female common carp invest significantly more (mean GSI = 0.227) into gonad per unit body mass than female bigmouth buffalo (mean GSI = 0.188), according to a *t*‐test comparing means (*t* = 2.66, df = 31, *p* = 0.0066, mean difference [±SE] = 0.0394 [0.0148]). Box and whisker plots are shown, with the large red bar indicating each sample mean.

Female common carp invest significantly more in reproductive tissue per unit body mass compared to bigmouth buffalo females. The pre‐spawn GSI of female common carp (*n* = 16) was on average 20.7% higher (common carp mean GSI = 0.227 versus bigmouth buffalo mean GSI = 0.188) than bigmouth buffalo (*n* = 17) (*t* = 2.66, df = 31, *p* = 0.0066, mean difference [±SE] = 0.0394 [0.0148]) (Figure 7).

### 3.3 Phenotypic Variation of Bigmouth Buffalo From Saskatchewan

**FIGURE 8:**
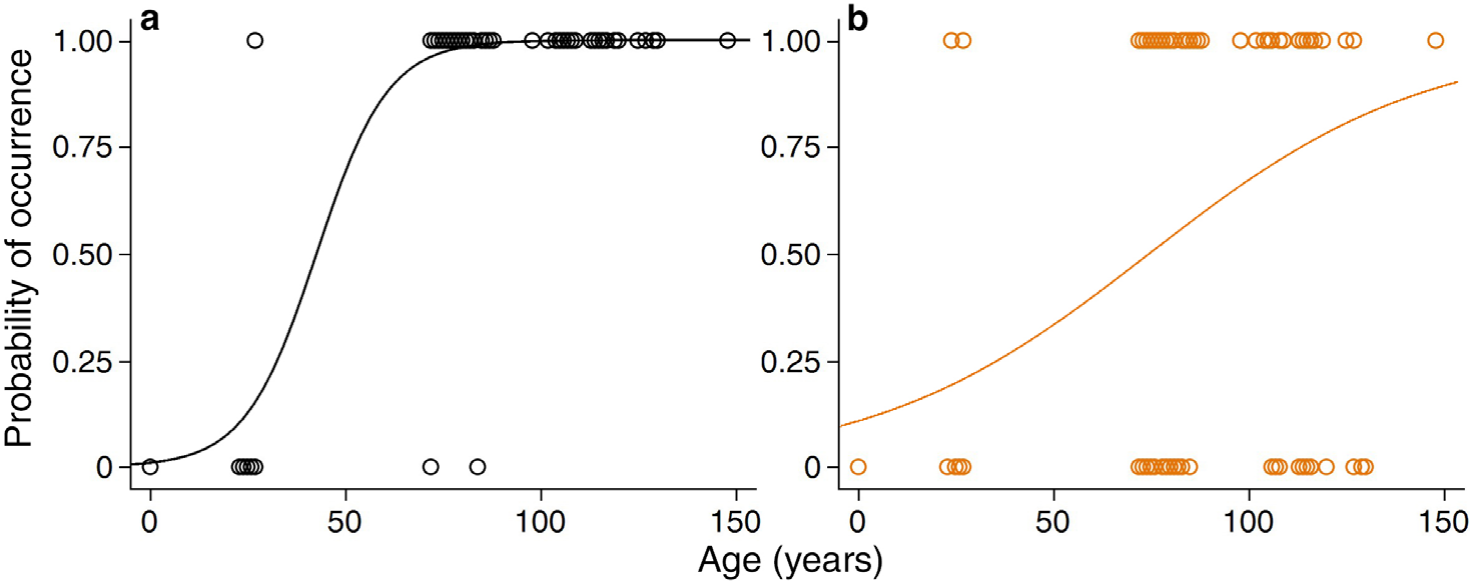
Likelihood of black or orange spots increases with age on bigmouth buffalo *Ictiobus cyprinellus* from Saskatchewan, Canada. (a) Logistic regression revealed that the presence of black spots increases in likelihood with bigmouth buffalo age (*χ* ^2^ = 152.4, df = 1, *n* = 169, *p* < 0.0001, *R* ^2^ = 0.83), and (b) that the presence of orange spots also increases in likelihood with age (*χ* ^2^ = 36.4, df = 1, *n* = 169, *p* < 0.0001, *R* ^2^ = 0.16). Data points represent the presence (1) or absence (0) of such markings. See Figure 9 (a–e, i, j, m–o) for examples of black and orange spots on bigmouth buffalo from Canada.

**FIGURE 9:**
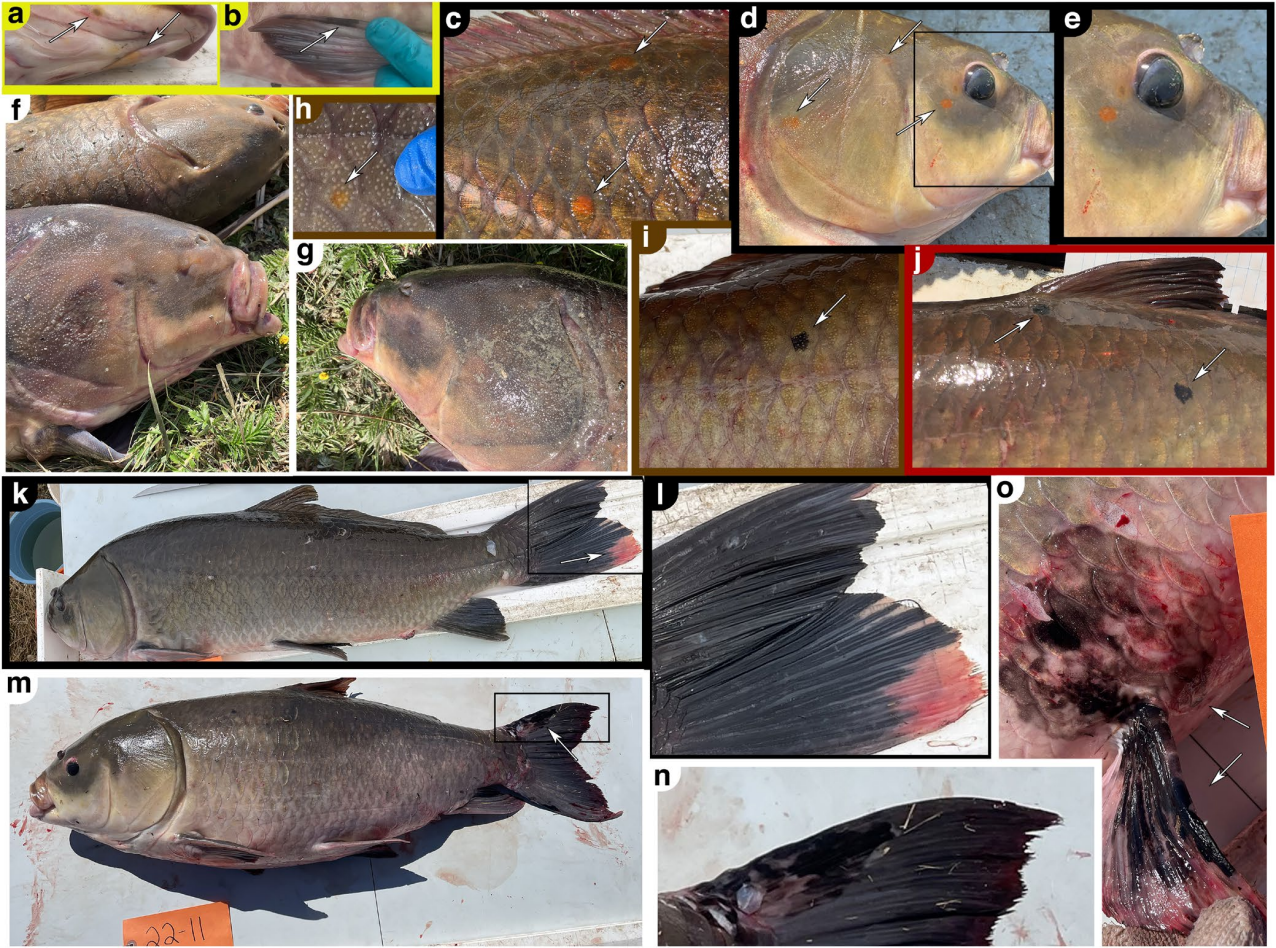
Morphological variation across eight individual bigmouth buffalo *Ictiobus cyprinellus* from Saskatchewan. (a, b) 109‐year‐old female with orange pigmentation spotting on its ventral area of head (a), and black pigmentation ridging on the leading fin ray of its right pelvic fin (b). (c–e) 106‐year‐old male with orange spots on its right side of body (c), and on right side of head (d) (with inset in e). (f, g) 108‐year‐old male that had no eyes. (h) 109‐year‐old male with an orange spot on a scale. (i) 80‐year‐old male with a black spot on its right side. (j) 79‐year‐old female with a couple black spots on its left side. (k, l) 75‐year‐old female with a white‐edged caudal fin (with inset in (l); see Figure 8e‐j in Lackmann, Seybold, et al. (2024)). (m–o) 116‐year‐old female with a large black spot on the dorsal margin of its caudal fin (with inset in n), and another large black spot on its right pelvic fin and body (o). See arrows (when present in a panel) for clarity.

Bigmouth buffalo from the Qu'Appelle system accrue black and orange pigmentation spots with age. Logistic regression analysis revealed that the presence of both black spots (*ꭓ* ^2^ = 152.4, df = 1, *n* = 169, *p* < 0.0001, *R* ^2^ = 0.83) and orange spots (*ꭓ* ^2^ = 36.4, df = 1, *n* = 169, *p* < 0.0001, *R* ^2^ = 0.16) increases in likelihood with age (Figure 8). According to these models, the age [±95% CI] at which black spots (e.g., Figure 9) become more likely to be present than absent on Saskatchewan bigmouth buffalo is 42.8 years [31.0, 52.4]; whereas for orange spots (e.g., Figure 9) this threshold is reached at 74.5 years [60.9, 87.0]. Additionally, 2 of 173 bigmouth buffalo (1.2%) from the Qu'Appelle system exhibited white‐edged fins (Lackmann, Seybold, et al. 2024), both of which were females in their 70s, and exhibited the white pigmentation (or depigmentation) in their caudal fin (e.g., Figure 9). Finally, one male bigmouth buffalo (108 years old) had no eyes nor evidence of eye sockets (Figure 9).

## 4 Discussion

The bigmouth buffalo is one of the longest‐lived vertebrates, which defies preconceived notions of the species. In a sample size of 173 individuals from Canada, we observe a maximum age of 148 years, which is 122 years older than their known maximum age prior to 2019 (Paukert and Long 1999; Lackmann et al. 2019). To our knowledge, the bigmouth buffalo is the 11th longest‐lived vertebrate species known (Table 2) among more than 66,000 species of extant vertebrates (IUCN 2014), which places bigmouth buffalo in the top 0.02% of vertebrate longevity. What is especially remarkable is that the bigmouth buffalo is not a cold‐adapted, deep‐dwelling, ocean‐adapted species like most other longest‐lived vertebrates (7 of 10; Table 2), but rather a warm‐adapted freshwater fish that thrives in shallow‐water habitats (Johnson 1963; Lackmann et al. 2019, 2021; Lackmann, Sereda, et al. 2023; Lackmann, Seybold, et al. 2024). Thus, we recommend the study of proximate mechanisms that may underlie the negligible senescence of bigmouth buffalo (Sauer et al. 2021), as these mechanisms (e.g., genetic underpinnings) are possibly novel.

**TABLE 2:** Review of the longest‐lived vertebrates and their maximum observed age, among the more than 66,000 species of extant vertebrates (IUCN 2014).

#	Common name	Scientific name	Age (years)	Structure/Method	Location	Ecosystem	Class	Source
1	Greenland shark	*Somniosus microcephalus*	392	Eye lens	Arctic Seas	Marine	Chondrychthyes	Nielsen et al. (2016)
2	Bowhead whale	*Balaena mysticetus*	211	Eye lens	Arctic Seas	Marine	Mammalia	George et al. (1999)
3	Rougheye rockfish	*Sebastes aleutianus*	205	Otolith	North Pacific (deep)	Marine	Actinopterygii	Cailliet et al. (2001)
4	Aldabra tortoise	*Aldabrachelys gigantea*	192	Captive*	Seychelles (originally)	Terrestrial	Reptilia	Reinke et al. (2022) and BBC (2024)
5	Galápagos tortoise	*Chelonoidis niger*	177	Captive	Galápagos (originally)	Terrestrial	Reptilia	Reinke et al. (2022)
6	Warty oreo	*Allocyttus verrucosus*	170	Otolith	Southern oceans (deep)	Marine	Actinopterygii	Stewart et al. (1995)
7	Shortraker rockfish	*Sebastes borealis*	160	Otolith	North Pacific	Marine	Actinopterygii	Kolora et al. (2021)
8	Black oreo	*Allocyttus niger*	153	Otolith	Southern oceans (deep)	Marine	Actinopterygii	McMillan et al. (2021)
9	Lake sturgeon	*Acipenser fulvescens*	152	Fin ray	Lake of the Woods, ON	Freshwater	Actinopterygii	Anonymous (1954)
10	Orange roughy	*Hoplostethus atlanticus*	149	Otolith	Deep oceans	Marine	Actinopterygii	Fenton et al. (1991)
11	Bigmouth buffalo	*Ictiobus cyprinellus*	148	Otolith	Last Mountain Lake, SK	Freshwater	Actinopterygii	This study

*Note:* *Individual alive as of 2025.

Abbreviations: ON, Ontario; SK, Saskatchewan.

The bigmouth buffalo is unique among animals as the only species that contains several populations known to have gone 40–50 years in between successful recruitment periods. For example, in the Pelican River watershed of northwestern Minnesota, multiple bigmouth buffalo recruitment gaps of 40 years or more exist across multiple lakes (Lackmann et al. 2019; *n* = 386). In east central Minnesota in the Rice River watershed of Rice Lake National Wildlife Refuge, a recruitment gap of 41 years exists between 1971 and 2012 with more than 95% of the extant population hatched from year classes before the year 1958, and 99.7% before 1972 (Lackmann, Seybold, et al. 2024, *n* = 390). In Arizona, no collected bigmouth buffalo younger than 85 years were observed in a sample size of 18 individuals (Lackmann, Black, et al. 2023). Finally, in the Qu'Appelle watershed of Saskatchewan spanning multiple sites and a variety of gear designed to collect the full range of fish size (inclusive of young‐of‐the‐year), a bigmouth buffalo (*n* = 173) recruitment gap of 48 years is evident between 1949 and 1997. To our knowledge, such extended periods with no evidence of recruitment, a phenomenon that recurs across diverse systems in bigmouth buffalo, are unparalleled in the animal kingdom. Bigmouth buffalo are a warm adapted species, and a potential hypothesis explaining episodic recruitment (or gaps in between recruitment) in the northern extent of their range is that short, cold growing seasons that prevent young‐of‐the‐year from reaching sufficient size to overwinter occur more frequently at higher latitudes. However, this hypothesis has not been tested, and other non‐mutually exclusive hypotheses could also explain recruitment variability. For example, predation on young‐of‐the‐year buffalofish (Lackmann, Seybold, et al. 2024), water level fluctuations in spring that affect the timing and duration of spawning or egg to fry survival (Lackmann, Sereda, et al. 2023), and drought intensity (Lackmann et al. 2021) could also vary with latitude.

Nonetheless, the ability of bigmouth buffalo to survive across decades without recruitment is enabled by their exceptional lifespan. We now know that bigmouth buffalo can live for nearly 150 years in habitats that induce highly episodic recruitment. This begs the question as to how broad recruitment gaps may be, or once were, before the oldest year class evident in each sample. For example, there is a more than 50‐year difference between the maximum observed age (96 years) at Rice Lake National Wildlife Refuge (*n* = 390), and the oldest known bigmouth buffalo (148 years). The highly episodic nature of bigmouth buffalo recruitment makes it challenging to understand the natural limit of their lifespan.

Bigmouth buffalo consistently achieve lifespans unparalleled by all other freshwater fish. Although the bigmouth buffalo is not technically the oldest‐living freshwater fish ever recorded (second to the lake sturgeon *Acipenser fulvescens* ; Table 2), the brochure that reported a 152‐year‐old lake sturgeon was written anonymously more than 70 years ago and was derived from a fin ray age estimate (Anonymous 1954). Fin rays are now well known to be unreliable indicators of fish age, especially in long‐lived species (Campana 2001; Radford et al. 2021). Furthermore, only otoliths have been age‐validated for long‐lived (> 50 years) lake sturgeon (Bruch et al. 2009), and lake sturgeon females estimated to be more than 80 years old are rare (Probst and Cooper 1954; WIDNR 2025) or nonexistent (Smith and Baker 2005; Bruch et al. 2009; Bauman et al. 2011). Moreover, lake sturgeon males are reported to have a maximum lifespan of 55 years (Bruch et al. 2009; USFWS 2016), though a maximum recorded age of 63 years has been predicted from a fin‐ray generated von Bertalanffy growth curve (Smith and Baker 2005). On the other hand, in each of the bigmouth buffalo populations previously mentioned, including several northwestern Minnesota lakes, east central Minnesota, Arizona, and Saskatchewan lakes, the median age is more than 80 years old as of 2025 and the populations are composed of numerous old‐age (> 90 years old) males and females (Lackmann et al. 2019; Lackmann, Sereda, et al. 2023; Lackmann, Black, et al. 2023; Lackmann, Seybold, et al. 2024). In addition, many supercentenarian male bigmouth buffalo are evident from Saskatchewan, including a 130‐year‐old (Figure 6), which is the oldest recorded male freshwater fish. To our knowledge, there is no other freshwater organism besides bigmouth buffalo that consistently achieves lifespans of more than 80 years, except (presumptively) some of the longest‐lived freshwater mussels (Unionida) (Ziuganov et al. 2000; Anthony et al. 2001).

Multiple lines of evidence indicate the bigmouth buffalo is declining amidst increased abundance of invasive common carp in the Qu'Appelle watershed of Saskatchewan. Perhaps most strikingly, there has been only one evident successful bigmouth buffalo year class since common carp were first detected in the watershed in 1955 (Figure 4; Atton 1959). Moreover, more than 75% of recruited common carp captured in the system are from year classes in the 2000s. Although there was significantly more bigmouth buffalo recruitment prior to the invasion of common carp (1955), this time coincides with the construction of Valeport Dam in 1958 and thus these two major changes to the ecosystem (in the past 150 years) are confounding. Nonetheless, not a single recruited bigmouth buffalo (0%) captured in the system was from a year class in the 2000s. Bigmouth buffalo were observed actively spawning during the spring of 2013 in Buffalo Pound Lake and in 2014 downstream of Last Mountain Lake (J. Sereda personal observations); however, more than a decade later, these cohorts are not evident in the population. Common carp recruitment was relatively stable in Last Mountain Lake, was positively correlated with spring water level in the post‐water control era (after 1958; Figure 5), and (in contrast to bigmouth buffalo) was not episodic. Furthermore, invasive common carp grow faster early in their ontogeny, exhibit an overall pace of life that is approximately three times more rapid, and females invest significantly more into reproduction (per unit body mass, per individual) compared to bigmouth buffalo (Figures 6 and 7; Table 1). Interestingly, the median age of these pre‐spawn female common carp was 13 years, versus 77 years for the pre‐spawn bigmouth buffalo (Figure 7). According to the observed maximum age of both common carp and bigmouth buffalo in this watershed, this equates to a GSI central tendency reference point at approximately 24.5% (13/53) and 52.0% (77/148) within the ontogeny of each species, respectively. Both being large‐bodied cypriniform fishes, it is surprising that common carp have already surpassed bigmouth buffalo in proportional reproductive investment two‐fold earlier within their ontogeny at this reference point, as evidenced through GSI. Like numerous other fishes, both common carp and bigmouth buffalo are known to become more fecund with increasing size and age (Weber and Brown 2012; Hixon et al. 2014; Barneche et al. 2018; Lackmann et al. 2021; Lackmann, Seybold, et al. 2024). Common carp are widely known to degrade freshwater ecosystems where they are non‐native (Weber and Brown 2012), but their potential for long‐term cryptic effects on long‐lived native freshwater fishes such as bigmouth buffalo is just being realized (Lackmann et al. 2025).

Bigmouth buffalo take longer to mature, can live for more than 110 years at approximate asymptotic length, and exhibit a more pronounced sexual‐size dimorphism than common carp in Saskatchewan. The von Bertalanffy growth models reveal that the instantaneous growth rate for bigmouth buffalo is approximately half that of common carp from the same lake (Table 1). Furthermore, female bigmouth buffalo from Last Mountain Lake are longer in mean asymptotic total length (*L*_*∞*_) than female common carp (Table 1). On the contrary, common carp males from the same lake exhibited an *L*_*∞*_ that was longer than bigmouth buffalo males (although 95% CIs were overlapping in this case; Table 1). This difference in the sex‐specific adult size differential between these two species indicates bigmouth buffalo are more sexually‐size dimorphic than common carp (Figure 6), which is expected (Winemiller 1992) given the differences in lifespan, growth, and maturation rates of these two species. Although large cyprinids are still generally considered “periodic” life history strategists (Winemiller 1992), common carp fall much closer on the triangular life history continuum to “opportunistic” life history strategists than do bigmouth buffalo. Thus, in theory (Winemiller 1992), one would expect the sexual size dimorphism of common carp to be less than it is for bigmouth buffalo (but females still larger than males in both species). Indeed, this is the case. In addition, Winemiller and Rose (1993) postulated that such evolutionary differences in life history manifest in other factors that regulate population dynamics, including recruitment. In the Qu'Appelle watershed of Canada, modifications to hydrology (e.g., dams), have increased the size of reservoirs and have affected the stability of the water levels (Lackmann, Sereda, et al. 2023). Modifications that stabilize hydrology may favor the population dynamics of moderately periodic species like common carp that fall much closer to the opportunistic side of the life history continuum, compared to extreme periodic life history strategists like bigmouth buffalo that evolved under environmental patchiness at broad scale. This should be further explored.

Bigmouth buffalo from Saskatchewan accrue black and orange spots that are comparable to other bigmouth buffalo populations assessed in North America. However, these black and orange spots appear at significantly older ages in Canada (Figures 8 and 9). The age at which the presence of black spots becomes more than 50% likely was approximately 43 years and was older than populations assessed in northwestern Minnesota (38 years) (Lackmann et al. 2019). The age at which the presence of orange spots becomes more than 50% likely was approximately 75 years in Saskatchewan, which was also older than found in northwestern Minnesota (42 years) (Lackmann et al. 2019). Comparing individuals greater than 70 years old across regions, the proportion of individuals with black spots from Canada was 98.5% (*n* = 130), whereas it was 100% (*n* = 186) from northwestern Minnesota (Lackmann et al. 2019), 100% (*n* = 325) from east central Minnesota (Lackmann, Seybold, et al. 2024), and 100% (*n* = 18) from Arizona (Lackmann, Black, et al. 2023). Comparing orange spots on individuals greater than 70 years old, prevalence was 62.3% (*n* = 130) from Canada, 98.9% (*n* = 186) from northwestern Minnesota (Lackmann et al. 2019), 59.1% (*n* = 325) from east central Minnesota (Lackmann, Seybold, et al. 2024), and 83.3% (*n* = 18) from Arizona (Lackmann, Black, et al. 2023). We hypothesize that these differences may be due to latitudinal variation in ultra‐violet light exposure, differences in habitat (e.g., turbidity), or diet (e.g., plankton species consumed). If orange or black spots evolved as a signal to conspecifics of vitality in advanced age, the delayed onset of these spots in Canada may reflect the longer time it takes individuals in this region to reach maturity and approximate asymptotic sizes. We also found that the proportion of bigmouth buffalo with white‐edged fins (Figure 9) was 1.2% (2 of 173) in Saskatchewan, which was comparable to the 3.3% (13 of 390) found in east central Minnesota (Lackmann, Seybold, et al. 2024). This phenotypic spot pigmentation variation across bigmouth buffalo in Saskatchewan, coupled with clear documentation and photos during capture and prior to the release of the fish, could help serve as individual identification markers in future tracking of old individuals across capture events spanning years (e.g., Lackmann, Black, et al. 2023), and potentially decades.

The bigmouth buffalo is one of the longest‐lived vertebrates, a quintessential periodic strategist (Winemiller and Rose 1992), and in decline across several populations (Eddy and Underhill 1974; Lackmann et al. 2021; Lackmann, Sereda, et al. 2023; Lackmann, Seybold, et al. 2024; Bryshun et al. 2025). This complex life history presents numerous challenges for fisheries management (Lackmann et al. 2019, 2021, 2025; Lackmann, Sereda, et al. 2023; Lackmann, Black, et al. 2023; Lackmann, Seybold, et al. 2024; Gutowsky et al. 2024; Bryshun et al. 2025), many of which are being recognized (Kopf et al. 2025). However, we emphasize two findings that have direct application for developing sustainable management strategies for bigmouth buffalo and other long‐lived fishes across their range, especially in the United States where bigmouth buffalo and other long‐lived fishes are managed as a single stock with common carp in fishing regulations (Winter 2024).

First, evidence of recent bigmouth buffalo recruitment does not indicate future recruitment (or population) stability. On the basis of year class distributions emerging from past recruitment, a bigmouth buffalo population could have decadal periods in the future with no recruitment even as the current population successfully produces offspring, or the population could be longevity overfished before the loss of older age classes is detected (Beamish et al. 2006; Kopf et al. 2025). As bigmouth buffalo age demographics from the Qu'Appelle watershed during the 1950s demonstrate, a cohort of relatively young and small individuals from 1940s’–year classes would have been evident. Indeed, this was documented by researchers during this time (Johnson 1963). This may have created a false sense of security (in fisheries) regarding recruitment success as the decades progressed, evidenced by the multidecadal unregulated commercial harvest of bigmouth buffalo from the Qu'Appelle watershed from approximately 1950–1980, even as bigmouth buffalo were significantly declining and evidence indicates they did not successfully recruit again until 1997 and have yet since (Lackmann, Sereda, et al. 2023). Furthermore, evidence of common carp recruitment has been successful and regular since 1970 in the Qu'Appelle watershed, contradicting assumptions that both populations could be managed as a single stock. Instead bigmouth buffalo management plans will need to account for the long lifespan, the episodic nature of their recruitment (i.e., the possibility that long recruitment gaps could occur), and the vulnerability to longevity overfishing (with potential negative impacts on other species and the broader ecosystem; Beamish et al. 2006; Kopf et al. 2025). Indeed, there have been other bigmouth buffalo populations assessed in sites from central and northern USA where commercial fishing occurs, where an absence of individuals more than 21 years old (Iowa, *n* = 115; Prull et al. 2023), more than 43 years old (Artichoke Lake, Minnesota, *n* = 52; Lackmann et al. 2019), and more than 58 years old is evident (North Dakota, *n* = 171; Lackmann et al. 2021). This is consistent with the hypothesis that longevity overfishing (Kopf et al. 2025) of bigmouth buffalo has already occurred in these populations.

Second, quantification of age score precision is essential for interpretation of year class (or age) distributions of bigmouth buffalo because the magnitude of precision in the age estimates affects the number of year classes estimated in a sample. Although otoliths have been age‐validated for the buffalofishes (Lackmann et al. 2019, Lackmann et al. 2021; Long et al. 2023), there is still precision (random error) that can be quantified (Campana et al. 1995; Campana 2001). For long‐lived fishes the coefficient of variation (CV) is recommended to quantify age score precision between multiple age readings because it is a robust, unitless metric that can be compared across studies (Campana et al. 1995; Campana 2001). Even if the random error is less than 5% between age readings (e.g., a median CV of 3.2% for bigmouth buffalo in this study), which is considered precise (Campana 2001), as ages become older this will generate a smoothing effect that spreads, or artificially scatters, year class estimates about modes (Campana 2001). For long‐lived fishes such as bigmouth buffalo that recruit episodically, this results in the number of year classes likely being overestimated in any given sample because year class peaks (modes) will be underestimated, while weaker year classes are overestimated due to this smoothing effect about true modes (Campana 2001). This is a direct result of the precision level of the age estimates, and the smoothing effect that it causes on year class (or age) distributions (Campana 2001).

Updated management plans are needed now to ensure there are future bigmouth buffalo populations in an era when most habitats and ecosystems have been and continue to be altered by humans. We have discovered that numerous bigmouth buffalo populations exhibit recruitment gaps longer than any other animal, which can be spread across their nearly 1.5‐century‐long lifespan. At the same time, several bigmouth buffalo populations in the northern extent of their range are significantly declining amidst increasing populations of invasive common carp. For these reasons, we recommend that the conservation status of bigmouth buffalo be elevated to direct the preservation of their populations while it is still possible.

## Author Contributions

**Alec R. Lackmann:** conceptualization (equal), data curation (equal), formal analysis (lead), investigation (lead), methodology (lead), resources (equal), software (equal), supervision (equal), validation (equal), visualization (equal), writing – original draft (lead), writing – review and editing (lead). **Jeff Sereda:** conceptualization (equal), data curation (equal), formal analysis (equal), funding acquisition (lead), investigation (supporting), methodology (supporting), project administration (lead), resources (equal), software (equal), supervision (lead), visualization (equal), writing – original draft (supporting), writing – review and editing (supporting). **James Villeneuve:** conceptualization (supporting), data curation (supporting), funding acquisition (supporting), investigation (equal), methodology (supporting), project administration (supporting), resources (supporting), software (supporting), supervision (supporting), visualization (equal), writing – review and editing (supporting). **Michelle Foley:** conceptualization (supporting), data curation (supporting), formal analysis (supporting), funding acquisition (supporting), investigation (supporting), methodology (supporting), project administration (supporting), resources (supporting), software (supporting), supervision (supporting), visualization (supporting), writing – review and editing (supporting). **Mike Pollock:** conceptualization (supporting), investigation (supporting), methodology (supporting), resources (supporting), software (supporting), visualization (supporting), writing – review and editing (supporting). **Reid Bryshun:** conceptualization (supporting), investigation (supporting), methodology (supporting), resources (supporting), software (supporting), visualization (supporting), writing – review and editing (supporting). **Katlin McCallum:** conceptualization (supporting), investigation (supporting), resources (supporting), visualization (supporting), writing – review and editing (supporting). **Ethan Englot:** conceptualization (supporting), investigation (supporting), resources (supporting), visualization (supporting), writing – review and editing (supporting). **Megan Zak:** conceptualization (supporting), data curation (supporting), investigation (supporting), methodology (supporting), project administration (supporting), resources (supporting), software (supporting), supervision (supporting), visualization (supporting), writing – review and editing (supporting). **Cole Rehbein:** formal analysis (supporting), investigation (supporting), resources (supporting), software (supporting), visualization (supporting), writing – review and editing (supporting). **Ewelina S. Bielak‐Lackmann:** investigation (supporting), methodology (supporting), resources (supporting), software (supporting), visualization (supporting), writing – review and editing (supporting). **Mark E. Clark:** conceptualization (supporting), formal analysis (supporting), investigation (supporting), methodology (supporting), resources (supporting), software (supporting), supervision (supporting), validation (supporting), visualization (supporting), writing – original draft (supporting), writing – review and editing (supporting).

## Disclosure

Impact Statement: The bigmouth buffalo exhibits recruitment gaps longer than any other animal and achieves lifespans approaching 150 years.

## Conflicts of Interest

The authors declare no conflicts of interest.

## Supporting information


**Appendix S1:** ece372483‐sup‐0001‐AppendixS1.docx.

**Data S1:** ece372483‐sup‐0002‐DataS1.xlsx.

**Data S2:** ece372483‐sup‐0003‐DataS2.xlsx.

## Data Availability

The data that support the findings of this study are provided as Supporting Information files.
