# Supplementary material for: Within‐Ecosystem Comparison of Bigmouth Buffalo Ictiobus cyprinellus and Common Carp Cyprinus carpio Reveals Diverging Population Trajectories, Declining Recruitment, and a Lifespan of 148 Years
Source: Ecol Evol. 2025 Nov 16;15(11):e72483. doi: 10.1002/ece3.72483 (PMC12620563; doi:10.1002/ece3.72483)
Supplement: Supplementary file 1 — Appendix S1: ece372483‐sup‐0001‐AppendixS1.docx. [file ECE3-15-e72483-s003.docx]

**Supplemental Material for:** Within-ecosystem comparison of bigmouth buffalo *Ictiobus cyprinellus* and common carp *Cyprinus carpio* reveals diverging population trajectories, declining recruitment, and a lifespan of 148 years

**Supplementary Tables**

**Table S1** Bigmouth buffalo *Ictiobus cyprinellus* von-Bertalanffy growth model selection statistics from the Qu’Appelle watershed, Saskatchewan.

| **Sample** | **Model** | ***n*** | **SSE** | **k** | **AICc** | **ΔAICc** | ***F*** | ***p*** | ***R*^2^** |
| --- | --- | --- | --- | --- | --- | --- | --- | --- | --- |
| Pooled | *k* NSS | 170 | 3007 | 3 | 496.6 | 0 | 3355.3 | <0.0001 | 0.976 |
| Pooled | Global | 170 | 3006 | 4 | 198.7 | 2.1 | 2223.9 | <0.0001 | 0.976 |
|  |  |  |  |  |  |  |  |  |  |
| BPL | *k* NSS | 89 | 1694 | 3 | 270.7 | 0 | 320.1 | <0.0001 | 0.883 |
| BPL | Global | 89 | 1677 | 4 | 272.0 | 1.3 | 213.3 | <0.0001 | 0.882 |
|  |  |  |  |  |  |  |  |  |  |
| LML | *k* NSS | 79 | 1148 | 3 | 220.0 | 0 | 713.2 | <0.0001 | 0.949 |
| LML | Global | 79 | 1148 | 4 | 222.3 | 2.3 | 469.2 | <0.0001 | 0.949 |

Footnotes: NSS = not sex-specific. SSE = sum of squares error; k = number of model parameters; AICc = Akaike’s Information Criterion corrected for small sample sizes; ΔAICc = Delta AICc; *F* = F statistic; *p* = P-value; *R*^2^ = coefficient of determination; BPL = Buffalo Pound Lake; LML = Last Mountain Lake

**Table S2** Common carp *Cyprinus carpio* von-Bertalanffy growth model selection statistics from the Qu’Appelle watershed, Saskatchewan. NSS = not sex-specific.

| **Sample** | **Model** | ***n*** | **SSE** | **k** | **AICc** | **ΔAICc** | ***F*** | ***p*** | ***R*^2^** |
| --- | --- | --- | --- | --- | --- | --- | --- | --- | --- |
| Pooled | Global | 119 | 3502 | 4 | 413.0 | 0 | 1247.5 | <0.0001 | 0.970 |
| Pooled | *k* NSS | 119 | 3571 | 3 | 413.1 | 0.1 | 1849.9 | <0.0001 | 0.970 |
|  |  |  |  |  |  |  |  |  |  |
| LML | *k* NSS | 83 | 1063 | 3 | 220.1 | 0 | 4059.1 | <0.0001 | 0.990 |
| LML | Global | 83 | 1062 | 4 | 222.3 | 2.2 | 2675.2 | <0.0001 | 0.990 |

Footnotes: SSE = sum of squares error; k = number of model parameters; AICc = Akaike’s Information Criterion corrected for small sample sizes; ΔAICc = Delta AICc; *F* = F statistic; *p* = P-value; *R*^2^ = coefficient of determination; LML = Last Mountain Lake

**Supplementary Figures**


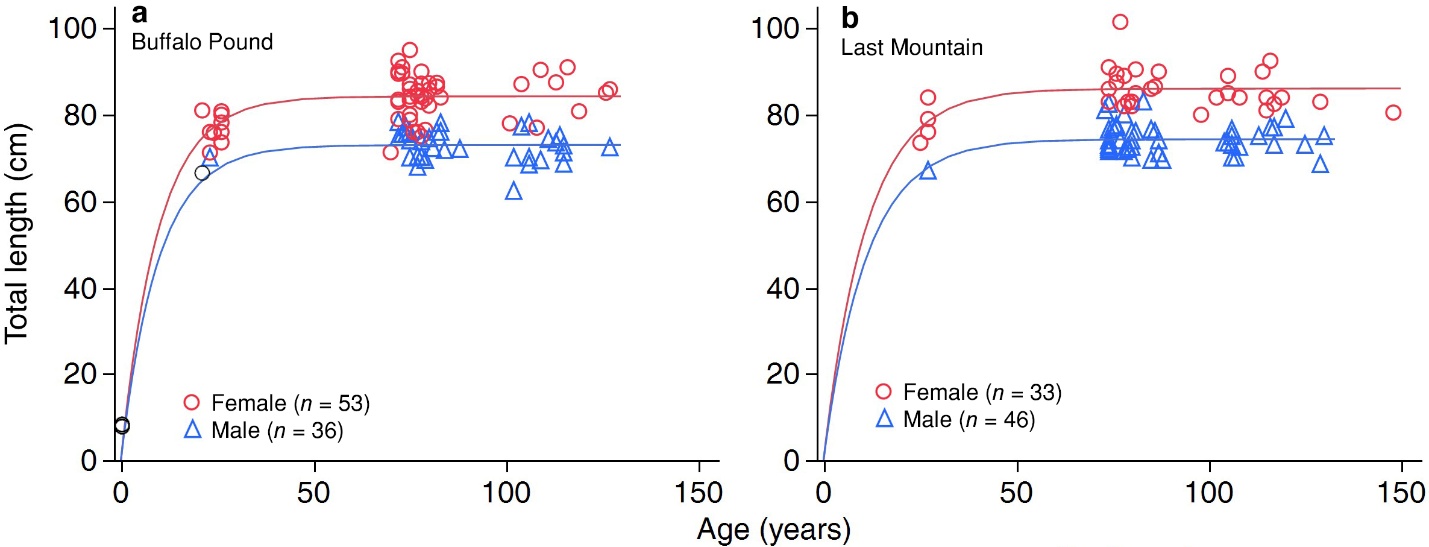


**Fig. S1** Total length versus age of bigmouth buffalo *Ictiobus cyprinellus* in two Canadian lakes. **a** Total length versus age of bigmouth buffalo from Buffalo Pound Lake, Saskatchewan (*F*_3,85_ = 320.1, df = 3, *p* < 0.0001, *R*^2^ = 0.88) with asymptotic length (*L_∞_*) for females of *L_∞_* = 84.3, 95% CI [82.9, 85.6] and males *L_∞_* = 73.0 [71.0, 75.0], growth rate (*k*) was *k* = 0.103 [0.087, 0.128], and the age at 0 length parameter [*t*_0_] was constrained by 1-month-old fry (see Methods). Unsexed individuals (open black circles; *n* = 3) are plotted for clarity, but did not contribute to the model. **b** Total length versus age of bigmouth buffalo from Last Mountain Lake, Saskatchewan (*F*_3,75_ = 713.2, df = 3, *p* < 0.0001, *R*^2^ = 0.95) with asymptotic length (*L_∞_*) for females of *L_∞_* = 86.1, 95% CI [84.6, 87.5] and males *L_∞_* = 74.4 [72.5, 76.2], growth rate (*k*) was *k* = 0.089 [0.075, 0.113], and the age at 0 length parameter [*t*_0_] was constrained by 1-month-old fry (see Methods). Points are coded by sex (red line = female model, blue line = male model). See **Table S1** for model selection statistics.


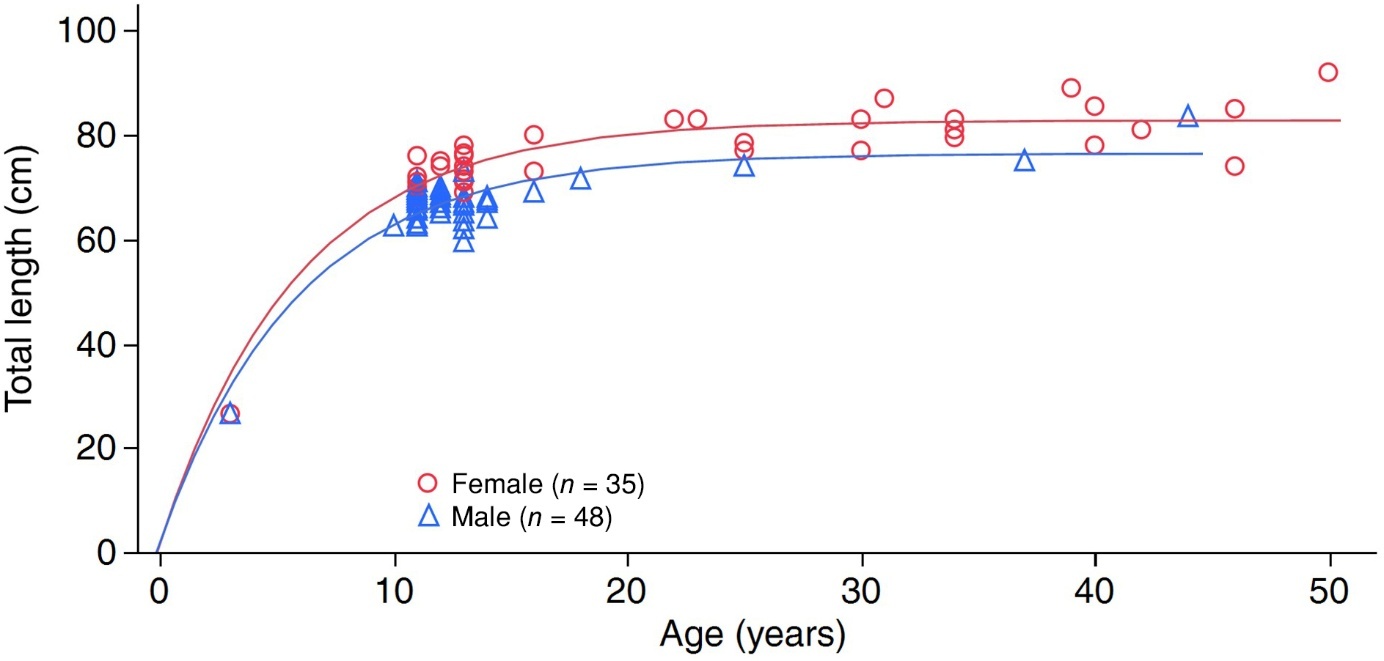


**Fig. S2** Total length versus age of common carp *Cyrinus carpio* from Last Mountain Lake, Saskatchewan (*F*_3,79_ = 4059.1, df = 3, *p* < 0.0001, *R*^2^ = 0.99) with asymptotic length (*L_∞_*) for females of *L_∞_* = 82.8, 95% CI [81.1, 84.5] and males *L_∞_* = 76.4 [74.4, 78.4], growth rate (*k*) was *k* = 0.170 [0.155, 0.188], and the age at 0 length parameter [*t*_0_] was constrained by 2-month-old fry (see Methods). Points are coded by sex (red line = female model, blue line = male model). See **Table S2** for model selection statistics.
